# Suppression of Urinary Voiding by Conditional High Frequency Stimulation of the Pelvic Nerve in Conscious Rats

**DOI:** 10.3389/fphys.2018.00437

**Published:** 2018-04-30

**Authors:** Charly B. J. Brouillard, Jonathan J. Crook, Pedro P. Irazoqui, Thelma A. Lovick

**Affiliations:** ^1^School of Physiology, Pharmacology and Neuroscience, University of Bristol, Bristol, United Kingdom; ^2^Weldon School of Biomedical Engineering, Purdue University, West Lafayette, IN, United States

**Keywords:** pelvic nerve, high frequency stimulation, conditional stimulation, urinary voiding, conscious rat

## Abstract

Female Wistar rats were instrumented to record bladder pressure and to stimulate the left pelvic nerve. Repeated voids were induced by continuous infusion of saline into the bladder (11.2 ml/h) via a T-piece in the line to the bladder catheter. In each animal tested (*n* = 6) high frequency pelvic nerve stimulation (1–3 kHz, 1–2 mA sinusoidal waveform for 60 s) applied within 2 s of the onset of a sharp rise in bladder pressure signaling an imminent void was able to inhibit micturition. Voiding was modulated in three ways: (1) Suppression of voiding (four rats, *n* = 13 trials). No fluid output or a very small volume of fluid expelled (<15% of the volume expected based on the mean of the previous 2 or 3 voids). Voiding suppressed for the entirety of the stimulation period (60 s) and resumed within 37 s of stopping stimulation. (2) Void deferred (four rats, *n* = 6 trials). The imminent void was suppressed (no fluid expelled) but a void occurred later in the stimulation period (12–44 s, mean 24.5 ± 5.2 s after the onset of the stimulation). (3) Reduction in voided volume (five rats, *n* = 20 trials). Voiding took place but the volume of fluid voided was 15–80% (range 21.8–77.8%, mean 45.3 ± 3.6%) of the volume expected from the mean of the preceding two or three voids. Spontaneous voiding resumed within 5 min of stopping stimulation. Stimulation during the filling phase in between voids had no effect. The experiments demonstrate that conditional high frequency stimulation of the pelvic nerve started at the onset of an imminent void can inhibit voiding. The effect was rapidly reversible and was not accompanied by any adverse behavioral side effects.

## Introduction

Neuromodulatory approaches have great potential for treating dysfunctional control of the urinary bladder, in particular urge urinary incontinence (UUI) where sufferers experience a “sudden and overwhelming desire to void, which is difficult to defer” (Abrams et al., 2003). In the clinical setting, sacral nerve stimulation is the procedure offered most commonly in cases that are refractory to current pharmacological and chemodenervation approaches, although other targets are also being used (see Janssen et al., 2017 for a recent review). In animal studies the net has been cast wider and there are several indications from studies in anesthetized cats and rats that stimulation of the tibial, saphenous, pudendal, dorsal penile, dorsal clitoral, and pelvic nerves all have the potential to modulate voiding (Snellings and Grill, 2012; Su et al., 2012a,b; Kovacevic and Yoo, 2014; Jen et al., 2016; Langdale et al., 2017; Moazzam and Yoo, 2017; Uy et al., 2017). In these studies, nerve stimulation was able to inhibit or reduce the frequency of voiding, or to decrease the rate of micturition-like contractions produced under isovolumetric conditions by producing an increase in bladder capacity.

**FIGURE 1:**
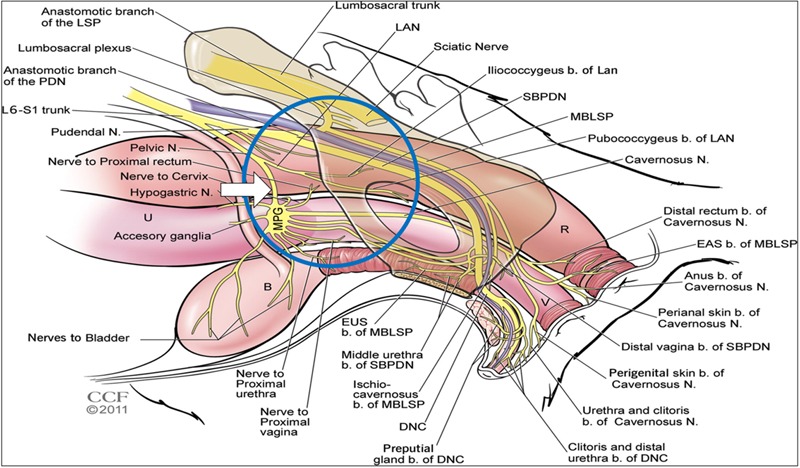
Diagram showing the anatomical arrangement of nerves innervating the pelvic viscera in the female rat. Blue circle shows area of interest. White arrow shows location of nerve cuff for stimulation. Reproduced from Pastelín et al. (2012) with permission from the copyright holder (John Wiley & Sons).

The aforementioned studies utilized continuous stimulation paradigms. An alternative approach would be to develop a conditional stimulation paradigm whereby the stimulus is applied only at the moment of need. Such an approach could be attractive in cases of UUI. In a recent study in anaesthetized rats, we stimulated the pelvic nerve immediately proximal to the pelvic ganglion (**Figure 1**). At this level, it contains predominantly bladder afferents, preganglionic parasympathetic efferent fibers, and some postganglionic sympathetic fibers (Hulsebosch and Coggeshall, 1982). We showed that voiding could be suppressed completely when high frequency stimulation was applied at the onset of an imminent void (Crook and Lovick, 2017). These findings in anesthetized preparations raised the possibility of developing conditional stimulation of the pelvic nerve as an alternative neuromodulatory approach to control UUI. As the next step toward this goal we carried out a study in conscious rats to determine whether stimulation of the pelvic nerve could modulate voiding.

## Materials and Methods

The study was approved by the University of Bristol Animal Welfare and Ethics Review Body and carried out under the authority of UK Home Office Project Licence PPL 30/3200. Female Wistar rats (Charles River, 254–315 g at start of the study, approximate age 11–15 weeks, estimated from supplier’s growth chart) were used in preference to males because surgical access to the pelvic nerve is easier in females. The rats were maintained on a 12-h on, 12-h off light schedule (lights on at 7 am) and had free access to standard rat chow and tap water.

### Pre-surgical Training

During the 10-day period prior to surgery, animals were habituated (3 × 30-min periods on separate days) to being confined in a Perspex restraining tube with a black nose cone in preparation for urodynamic measurements made later in the study. The rats were unable to turn round in the tube but were able to orientate their limbs into a natural posture and to groom their head region. Once fully habituated they entered the tube readily and once in place, remained quiet during the recording sessions apart from occasional bouts of grooming the head and/or sniffing. On two additional days during the pre-surgical period the rats were placed in a metabolic cage (Techniplast) for 24-h periods to measure the pattern of voiding. The apparatus was modified so that urine arriving in the collection chute drained into a covered container placed on the pan of a digital balance, in order to measure the timing and weight of voids.

### Electrodes and Bladder Catheters

Cuff electrodes were fabricated in-house from platinum iridium wire with a cobalt core embedded in silicone epoxy (Med-4213, Nusil, Carpinteria, United States) to the following dimensions: ID 0.5 mm, OD 1.2 mm, cuff length: 2 mm, inter-electrode distance: 1 mm, lead length: 200 mm (Crook et al., 2018). Bladder catheters were made from polyethylene tubing (ID = 0.76 mm; OD = 1.22 mm; length: 300 mm) with a “stop collar” at one end made by tying 6.0 suture thread round the tubing 5 mm from the tip. A short length of stainless steel tubing cut from a 21G needle was inserted into the opposite end of the catheter and capped with a short length of polythene tubing sealed at one end with a drop of silicone glue.

### Surgery

Surgery was performed under isoflurane anesthesia and under strict aseptic conditions. Prior to making the first incision the rats received 5 ml saline s.c. and 15 μg of buprenorphine analgesic (Vetergesic i.p. 0.3 mg/ml).

Areas of skin on the nape of the neck and abdomen were shaved and the skin cleaned with 2% Hibitane and 70% ethanol. A small transverse incision was made at the nape of the neck (20 mm) and a longitudinal midline incision (60 mm) was made in the abdomen. A trochar was tunneled subcutaneously via the right flank to connect the abdominal and nuchal openings, to allow the bladder catheter and leads from the pelvic nerve electrode cuff to be passed through. A midline laparotomy was performed and the abdominal wall retracted. The bladder was deflected to the animal’s right side in order to expose the left pelvic nerve, which was located running along the lateral wall of the uterus posterior to the ureter. A length of the postganglionic nerve bundle was carefully separated from the wall of the uterus for about 2 mm proximal to the pelvic ganglion without disturbing the nearby nerves that emanate from the ganglion (**Figure 1**). A 6.0 silk suture thread was looped under the nerve and the cuff electrode slipped under the nerve and closed to achieve a snug, but not tight, fit by tying the suture thread round the nerve cuff (Crook et al., 2018). A drop of dental silicone (Klasse 4) was used to cover the nerve-cuff assembly.

The bladder was emptied of urine via a 23G needle inserted through the bladder dome. The saline-filled catheter was then inserted into the bladder through the same incision. The “stop collar” prevented the catheter from advancing too far into the bladder. The incision was closed using a purse string suture, which was anchored to the stop collar. The assembly was sealed with a drop of tissue glue (Vetbond, 3Ms). The abdominal incision was then closed with a continuous Vicryl suture (Ethicon) with the catheter and leads from the pelvic nerve cuff arranged to exit the incision at the rostral end. Excess catheter tubing and leads were loosely sutured to the abdominal wall at 2 points. The skin incision was then closed using wound clips (Michel Suture Clips, 12 × 2.5 mm, Fine Science Tools, Heidelberg, Germany).

The rat was turned prone. The distal end of the bladder catheter was passed through a small hole drilled in the rim of a skin button (PlasticsOne) and sealed in place using superglue and dental acrylic cement (Simplex Rapid, Kemdent). The gold pins attached to the electrode leads were slotted into the sockets in the skin button and sealed in place with cement. Then, a 2-cm diameter circular piece of polypropylene non-absorbable mesh (Premilene^®^ Mesh, B Braun Surgical) was tied to the brim of the skin button to form a flange, which was sutured to the internal layer of the dermis. Finally, the skin incision was closed with silk sutures. A screw-on dust cap (PlasticsOne) protected the electrode terminals when not in use.

Once the surgery was complete, antibiotic (Amoxicillin, 15 mg/kg) was administered subcutaneously and the skin round the wound area was painted with a bitter solution (STOP ’n GROW, The Mentholatum Co., Ltd.) to discourage rats from chewing the wound clips. The animals were placed in a warm environment to recover from anesthesia.

Rats were monitored closely over the next few days to ensure that they were eating and drinking normally and to check that voiding had resumed. The bladder catheter was flushed daily (0.3 ml saline over 10 s). Four to eight days after surgery the rats were placed in the metabolic cage for 24 h to check that normal voiding patterns had re-established.

### Experimental Protocol

Experiments were started 8–13 days following surgery and continued for up to 79 days post-surgery (**Table 1**). Rats were placed in the confinement tube, which allowed fluid voided by the rat to be collected in a beaker on the pan of an electronic balance placed under the restraining tube. Generalized movements of the rat were detected by a Flexible PVDF Piezo device (Measurement Specialties) taped to the rat’s tail. A spirometer (AD Instruments) placed at the end of the nose cone of the restraining tube was used to monitor air movements produced by sniffing activity and movements of the rat’s head. The outputs from these devices were integrated over 1-min periods to provide an indication of general activity of the rat inside the restraint tube.

**Table 1:** Summary of experimental condition for individual rats.

Rat	1	2	3	4	5	6
Duration of experiment (days from surgery to final experimental session)	30	15	79	70	70	13
Total experimental sessions	5	3	18	12	15	3
% of sessions when pelvic nerve stimulated	40%	33%	72%	42%	40%	33%
% of stimulation sessions when voiding inhibited	100%	100%	100%	100%	83%	100%
Total number of stimulations (all sessions)	5	1	22	9	6	1
% of stimulations that inhibited voiding	60%	100%	91%	89%	100%	100%

In the conscious rats, pelvic nerve stimulation was delivered via a constant current stimulator (STMISOLA, Biopac System Inc.). A sinusoidal waveform was used to deliver a charge balanced stimulus and to be consistent with our previous study in terminally anesthetized rats. Saline was infused into the bladder at 11.2 ml/h using a syringe pump (model “22” MA1 55-2222, Harvard Apparatus). A T-piece in the line allowed simultaneous measurement of bladder pressure via a pressure transducer (MLT0699, AD instruments) and amplifier (BP Amp FE117, AD Instruments). Data was captured using a Powerlab 8sp data acquisition system (AD Instruments) operating in Chart mode. In each experiment, a period of 10 min was allowed for the rat to settle once it had been introduced into the restraining tube. Saline was then infused into the bladder at 11.2 ml/h.

### Terminal Experiments Under Anesthesia

In six rats terminal experiments under urethane anesthesia (1.4 g/100 g i.p.), were carried out 8–10 weeks after surgery for chronic implantation of devices, following the protocol described in Crook and Lovick (2017). In brief, the anesthetized animal was positioned supine, the abdomen was opened, and insulated platinum wire electrodes were placed under the pubic symphysis to record EMG activity of the external urethral sphincter (EUS). Pelvic nerve stimulation was delivered via the implanted nerve cuff electrode. At the end of the experiment, animals were killed with an overdose of anesthetic.

### Statistical Analysis

Data is expressed as mean values ± SEM. Most of the data were analyzed using paired Student’s *t*-tests except when the distribution was not normal, in which case Wilcoxon signed-rank test was used. A repeated measures ANOVA with a Tukey *post hoc* test was used to compare the volume voided during the pelvic nerve stimulation with the pre-stimulation and post-stimulation spontaneous voids. All the data were analyzed using Graph Pad Prism v.7 software.

## Results

### Diurnal Pattern of Urine Output

**FIGURE 2:**
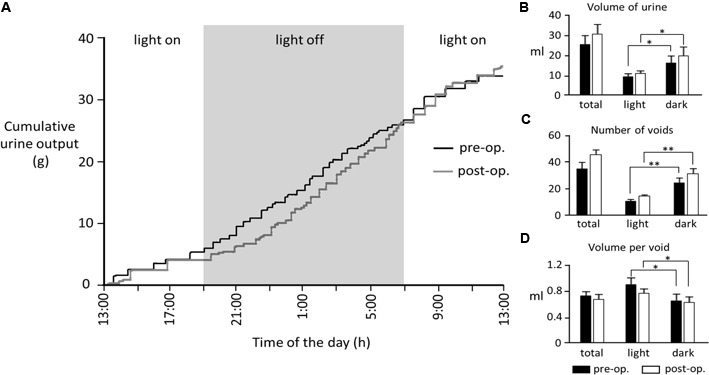
**(A)** Cumulative 24-h urine output from a rat measured 6 days prior to and 4 days post-surgery. **(B–D)** Bar graphs show pooled data from five rats. In the remaining animals, 24-h data sets were incomplete due to computer crashes during data acquisition. ^∗^*p* < 0.05, ^∗∗^*p* < 0.01 (Wilcoxon signed-rank test).

Nine rats (body weight 254–315 g, mean 277 ± 10 g) were implanted with bladder catheters and pelvic nerve cuff electrodes. All rats initially recovered well. However, data from three rats was later discarded (see below). When tested 4–8 days after surgery, the remaining six animals displayed a diurnal pattern of urine output characterized by more frequent voids during the dark period compared to the light (**Figure 2**). There was considerable inter-animal variation in the volume of urine produced over 24 h (12.7–36.2 ml); however, there was no significant difference in pre- and post-operative values (mean 23.3 ± 4.0 ml vs. 26.6 ± 5.1 ml, respectively, *p* = 0.12, Wilcoxon signed-rank test, *n* = 5).

### Urodynamic Studies

**FIGURE 3:**
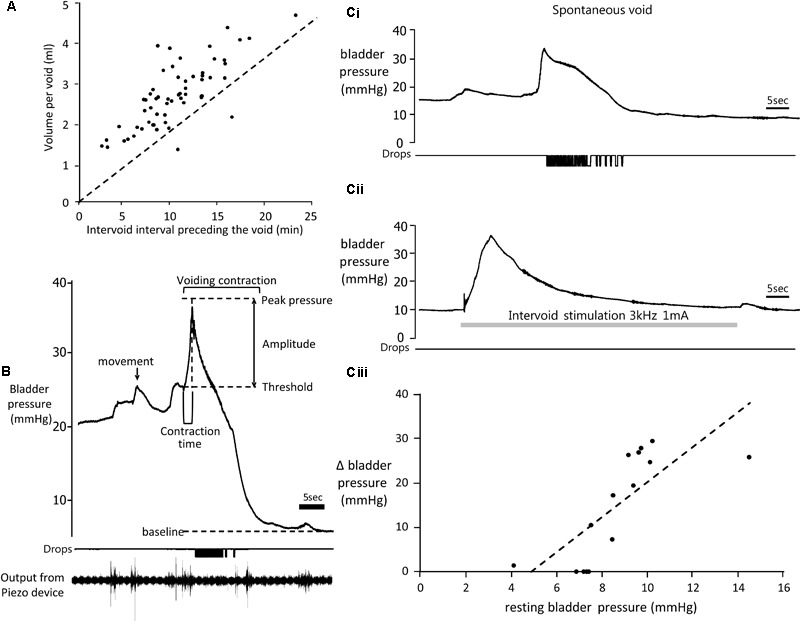
**(A)** Relationship between voided volume and inter-void interval (time elapsed since the previous void). Broken line shows expected voided volume based solely on volume of saline infused over same time period. Data from six rats. **(B)** Top trace: Example of change in bladder pressure during a void induced by infusion of saline into the bladder. Trace annotated to show urodynamic measurements made. Middle trace: timing of drops of urine voided. Lower trace: output from the piezo device detecting gross movements of the animal. **(Ci)** Change in bladder pressure during a spontaneous void during infusion of saline into the bladder (11.2 ml/h). **(Cii)** Effect of stimulation of the pelvic nerve (gray panel, 3 kHz 1 mA) in the same rat during the interval between voids. **(Ciii)** Relationship between amplitude of the response to pelvic nerve stimulation and resting bladder pressure (data from six rats).

Three rats failed to complete the study due to blockage of the bladder catheter (*n* = 2) or migration of the connections to the skin button (*n* = 1). Data from these rats was not included in the analysis. In the remaining six rats for which complete data sets were obtained, infusion of saline into the bladder (11.2 ml/h) evoked repeated voiding. There was considerable variation in inter-void interval both between and within animals (2.5–17.5 min, mean 10.17 ± 0.5 min). Typically, the volume of fluid voided after a long inter-void interval was greater than after a short interval (**Figure 3A**) and exceeded the volume of saline infused, presumably reflecting output of urine from the kidneys draining into the bladder via the ureters.

#### Characteristics of Bladder Pressure Changes During Continuous Infusion Cystometry

Resting bladder pressure at the beginning of an experiment prior to starting infusion of saline ranged from 2.8 to 7.2 mmHg, mean 4.54 ± 0.49 mmHg (*n* = 28 testing sessions in 6 rats). Resting pressure varied in the same rat on different days, presumably depending on the extent of bladder fullness at the beginning of the experiment. At the start of the infusion, there was little change in bladder pressure until approximately 1.5 ml of saline had been infused. Above this volume, the pressure started to rise more steeply. Just before a void occurred the rats became noticeably more restless in the tube and oscillations were detected in the bladder pressure record. Movements of the animal detected by the piezo device on the tail often corresponded in time with oscillations in pressure, suggesting that they largely reflected changes in intra-abdominal pressure transmitted to the bladder when the animal moved (**Figure 3B**).

**FIGURE 4:**
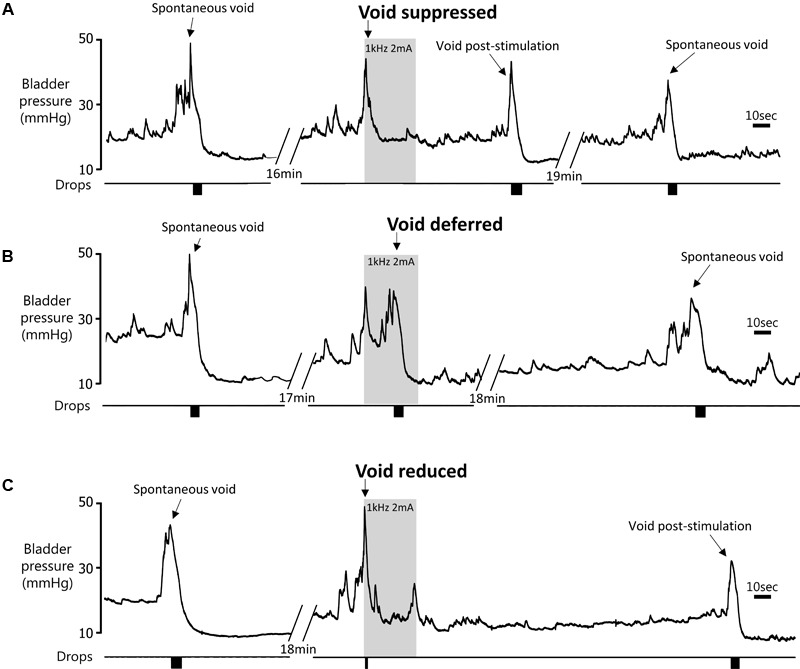
Effect of 60 s pelvic nerve stimulation applied at the onset of an imminent void on void volume during the stimulation period. Voids were either suppressed during the stimulation period **(A)**, initially suppressed and deferred until later in the stimulation period **(B)**, or a void took place but the volume was reduced compared to pre-stimulation **(C)**.

Voids were preceded by a sharp rise in bladder pressure (**Figures 3B,Ci**). Bladder pressure increased from a threshold pressure of 19.8 ± 0.6 mm Hg (mean of 102 spontaneous voids in the absence of pelvic nerve stimulation) reaching a peak after 2.8 ± 0.2 s. Although void threshold pressure could vary in the same rat on different days, it remained relatively constant during each experiment. Bladder contraction pressure (difference between threshold and peak pressures) was 18.7 ± 1.1 mmHg (*n* = 102). In some rats the bladder pressure plateaued after reaching the peak (**Figure 3Ci**), before falling rapidly as fluid was voided. On other occasions the contraction was not sustained and pressure reduced rapidly back to baseline (**Figure 4B**). Both types of response could be observed in the same animal.

#### Response to Stimulation of the Pelvic Nerve in Between Voids

We used the first recording session following surgery to make baseline urodynamic measurements and establish that infusion of saline into the bladder evoked repeated voids. We also stimulated the pelvic nerve in between voids in order to observe whether it evoked any behavioral responses and to determine if stimulation would influence bladder status. Stimulation parameters tested (1–2 mA, 1–3 kHz sinusoidal stimulation) were shown previously by us to be optimal for inhibiting voiding in anesthetized rat preparations (Crook and Lovick, 2017).

None of the rats showed any signs of discomfort, distress or restlessness during stimulation using these intensities. No fluid was released from the bladder during stimulation. In most cases (9/14 tests) a transient increase in bladder pressure was evoked at stimulus onset (**Figure 3Cii**), which was of similar magnitude to the rise in pressure that occurred during spontaneous voids (**Figure 3Ci**). However, the time course of the stimulation-evoked pressure rise was much slower (time to peak pressure 4.1 ± 1.2 s) compared to during the preceding spontaneous voiding contraction (2.4 ± 0.5 s, *n* = 9, *p* < 0.01 paired *t*-test). The magnitude of the stimulation-evoked increase in bladder pressure also depended on resting pressure. Stimulating the pelvic nerve when the bladder was approaching fullness and intravesicular pressure had started to rise, evoked a bigger response compared to stimulating when bladder pressure was close to basal level (**Figure 3Ciii**). Visual inspection of the rat revealed a transient contraction of the abdominal wall at the start of the stimulation period. In 3/14 tests a single fecal pellet was expelled during stimulation.

#### Response to Stimulation of Pelvic Nerve When a Void Was Imminent

The primary object of this study was to investigate whether pelvic nerve stimulation could modulate imminent urinary voiding in conscious rats. Due to legal constraints on the length of time we were permitted to keep rats confined in the holding tube during an experiment, in practice only two stimulation trials were usually possible in a single session. We therefore chose to restrict stimulation to within the range of parameters that we had found previously to be optimal for inhibiting voiding in anesthetized rats (0.5–5 mA, 1–3 kHz sine wave; Crook and Lovick, 2017). On all other occasions we restricted stimulation parameters to 2 mA or less. Higher intensities (3 or 4 mA, *n* = 2) elicited signs of discomfort (struggling and/or vocalization) and were not repeated.

**FIGURE 5:**
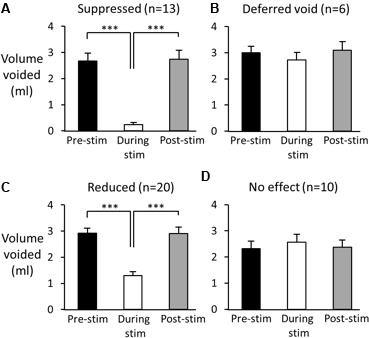
Effect of high frequency stimulation of the pelvic nerve on voiding. Black bars show mean volume of voids prior to stimulation. Open bars: volume of urine voided during pelvic nerve stimulation; grey bars volume of first void post stimulation. **(A–C)** Examples of suppressed **(A)**, deferred **(B)**, and reduced voiding **(C)**. In each case normal voiding resumed once the stimulation ceased. **(D)** On 10 further occasions the stimulus was ineffective. Data from 49 stimulation tests in six rats. ^∗∗∗^*p* < 0.001 (repeated measures ANOVA, Tukey’s *post hoc* test).

In each rat (6/6 animals tested) initiating pelvic nerve stimulation at the onset of the steep rise in bladder pressure signaling an imminent void was able to inhibit the void. Voiding was modulated in three ways:

(1) Suppression of voiding (seen in 13 tests in four rats) – no fluid output during the 60-s stimulation period or a very small volume of fluid expelled (<15% of the volume expected based on the mean of the previous two or three voids; **Figures 4A,5A**).

(2) Voiding deferred (seen in six tests in four rats) – the imminent void was suppressed (no fluid expelled) but a void of similar volume to the mean of preceding voids occurred before the end of the stimulation period (12–44 s, mean 24.5 ± 5.2 s after the onset of the stimulation; **Figures 4B,5B**).
(3) Reduction in voiding (seen in 20 tests in five rats). Here, the imminent void proceeded but the volume of fluid voided was <80% of the volume expected from the mean of the preceding two or three voids (range 21.8–77.8%, mean 45.3 ± 3.6% of the previous voids; **Figures 4C,5C**).

**FIGURE 6:**
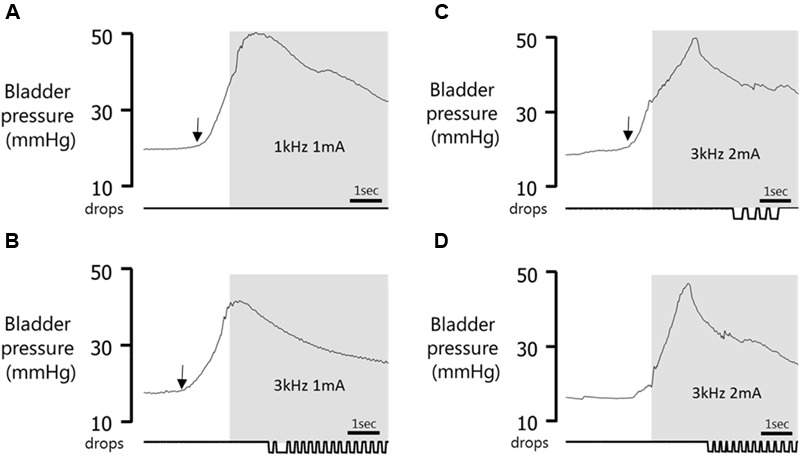
Effect of timing of stimulus onset. Gray panel shows stimulation period. **(A)** Void suppressed when stimulation was applied 1.13 s after the start of the bladder contraction. **(B)** No effect on voiding in same experimental session when stimulation was started 1.40 s after the onset of bladder contraction. **(C)** Void volume reduced (compared to prior spontaneous voids) when pelvic nerve stimulation was initiated 1.01 s after the start of the bladder contraction. **(D)** Stimulation in error during a spontaneous fluctuation in pressure when bladder nearing capacity induces a rise in bladder pressure and expulsion of urine. Arrow indicates onset of the bladder contraction.

In 10 additional tests in four rats, stimulation failed to alter the course of the void, despite stimulation using the same parameters being effective when tested later in the same session (**Figure 5D**). The cystometry record showed that in five of the ineffective cases, the delay in starting the stimulation after the onset of the steep rise in bladder pressure that precedes voiding was longer than when the same stimulation was effective in inhibiting voiding (1.9 ± 0.2 s vs. 1.4 ± 0.1 s, *n* = 5, *p* = 0.06 paired *t*-test; **Figures 6A,B**). In the other five cases close inspection of the cystometry record showed that stimulation had been initiated in error; these cases have been excluded from summary data **Table 1**). The bladder pressure had not begun the steep rise that signals an imminent void (**Figures 6C,D**). Interestingly, once the stimulation started, bladder pressure underwent a steep increase (**Figure 6D**), presumably reflecting the increase in intra-abdominal pressure that accompanies the transient abdominal contraction at the onset of pelvic nerve stimulation as shown in (**Figure 3B**). Since the bladder would likely have been close to capacity as judged from volume infused since the preceding void, urine may have been expelled forcibly rather than by a natural micturition. Measurements of intra-abdominal pressure and ideally EMG activity in the EUC will be required to resolve this issue.

#### Resumption of Voiding Post Stimulation

When a void had been suppressed by pelvic nerve stimulation, the next void occurred within 37.5 ± 14.1 s of terminating the stimulus and the volume was similar to pre-stimulus control voids (2.8 ± 0.35 ml pre-stimulation vs. 2.7 ± 0.3 ml post-stimulation, *p* > 0.05; **Figure 6A**). If a void had been deferred, i.e., initially suppressed but a void occurred before the end of the 60 s stimulation period, the next void took place within 787 ± 107 s of terminating the stimulus and the volume was similar to control pre-stimulation voids (respectively, 3.1 ± 0.3 ml vs. 3.0 ± 0.2 ml, *p* > 0.05). When the imminent void proceeded but the volume voided was reduced compared to the preceding spontaneous voids, the next void took place within 274 ± 50 s of terminating the 60 s stimulation and the volume was similar to pre-stimulation control voids (respectively, 2.8 ± 0.24 ml vs. 2.9 ± 0.2 ml, *p* > 0.05; **Figure 6C**).

#### Reproducibility

The effect of pelvic nerve stimulation was tested intermittently at intervals of 1–12 (mean 3.6 ± 0.4) days post-surgery. In three animals, regular testing was carried out for 4–5 weeks post-surgery, and then paused 2 weeks for two rats, and one rat for 4 weeks (researcher vacation period), although patency of the bladder catheter was maintained by regular flushing. In each of these rats, pelvic nerve stimulation was still effective when pelvic nerve stimulation was resumed 2–4 weeks later.

Although a total of 56 experimental sessions were undertaken, pelvic nerve stimulation was carried out in only 50% (**Table 1**). On some days, artifactual changes in bladder pressure, perhaps due to gut movements impacting on the bladder, made it difficult to determine when a void was imminent. So the effect of stimulation was not tested. On others, the infusion of saline failed to evoke regular voids although good voiding would be established in a subsequent session.

In different sessions the same stimulation might produce complete suppression, a reduction in voided volume or a delay in the onset of the imminent void. We were interested in understanding what determined which of these effects was evoked by the stimulation. We were unable to detect any relationship between type of response evoked and stimulation parameters within the relatively narrow range tested (*p* > 0.05 chi squared test for 1 kHz vs. 3 kHz; 0.5–1 mA vs. 2–4 mA). There was also no detectable relationship between generalized activity or sniffing behavior, time of day the tests were carried out (morning or afternoon), or the number of days since surgery and the stimulus outcome (*p* > 0.05 Kruskal–Wallis test; **Table 2**). In terms of urodynamic parameters, there was no correlation with response type and voiding pressure threshold, volume of fluid infused since the previous void, latency of stimulus onset with respect to start of bladder contraction or end fill compliance (*p* > 0.05 Kruskal–Wallis test; **Table 2**).

**Table 2:** Relationship between urodynamic and behavioral measures and effect of pelvic nerve stimulation on voiding.

	Void	Void Delayed	Void Reduced	*p* value
	Suppressed			Kruskal–
				Wallis test
Session start (hours since light on)	6.87 ± 0.51	8.08 ± 1.08	5.75 ± 0.38	0.094
Days post-Surgery	41.77 ± 6.91	31.00 ± 7.15	36.30 ± 5.24	0.646
Stimulus onset latency (s)	1.36 ± 0.16	1.21 ± 0.16	1.73 ± 0.17	0.116
Duration of bladder pressure rise (s)	2.89 ± 0.22	2.20 ± 0.38	3.27 ± 0.27	0.213
Bladder pressure threshold (mmHg)	19.88 ± 1.17	15.49 ± 2.77	18.73 ± 1.10	0.123
Void interval since the previous void (min)	12.32 ± 1.43	13.31 ± 1.50	12.73 ± 0.84	0.910
End-fill bladder compliance (ml/mmHg)	0.13 ± 0.01	0.17 ± 0.02	0.13 ± 0.01	0.129
Sniffing and head movement (mV/s)	2.02 ± 1.68	2.26 ± 4.88	-0.16 ± 3.28	0.675
Gross movement (V/s)	0.00 ± 0.04	0.03 ± 0.07	-0.09 ± 0.09	0.968

*Mean values ± SEM.*

### Off Target Effects

Pelvic nerve stimulation at 0.5–2 mA (intensities that inhibited voiding) never evoked any signs of distress. However, in all rats a brief contraction of the abdominal wall was clearly visible at the onset of stimulation. When the stimulation was applied between voids an increase in bladder pressure was seen to accompany the abdominal contraction (**Figure 4B**).

Defecation was observed during some experimental sessions but was infrequent and variable. The number of fecal pellets released during each experimental session varied from 0 to 5/h. Over 28 sessions in which stimulation was applied (approximately 50 h) only 32 pellets were expelled; 18 were expelled during a period of pelvic nerve stimulation.

### Terminal Experiments Under Anesthesia

**FIGURE 7:**
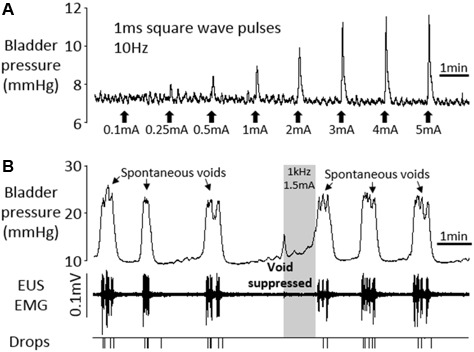
Terminal experiment under urethane anesthesia. **(A)** Graded increases in bladder pressure in response to 10 s periods of square wave pelvic nerve stimulation at 10 Hz, 1 ms pulses. **(B)** Voiding in response to continuous infusion of saline into the bladder (6 ml/h). High frequency pelvic nerve stimulation (1 kHz 1.5 mA sinusoidal waveform, gray panel) suppressed imminent spontaneous void.

In order to assess the functional integrity of the nerve–bladder connection after chronic implantation of electrodes on the pelvic nerve, we carried out a terminal experiment in six rats 3–12 weeks after surgery. The stimulation parameters used (10 s trains of 1 ms rectangular pulses at 10 Hz) were based on those we had already established to be optimal for evoking contraction of the bladder in urethane-anesthetized preparations (Crook and Lovick, 2017). In all rats, low frequency stimulation (10 Hz) evoked a graded increase in bladder pressure (**Figure 7A**). In four of six rats, continuous infusion of saline into the bladder (6 ml/h) evoked repeated voids, which were characterized by a sharp rise in bladder pressure and the appearance of bursting activity in the EMG recorded from the EUS (**Figure 7B**). The rise time of the bladder contraction recorded under anesthesia (7.7 ± 1.0 s, *n* = 24 voids) was significantly longer than in conscious rats (2.8 ± 0.2 s, *n* = 102 voids; *p* < 0.01, Student’s *t*-test). As in conscious animals, there was considerable inter-animal variability in inter-void interval (0.7–8.2 min) and voiding threshold pressure (12.2–20.0 mmHg).

In 3/4 animals, high frequency pelvic nerve stimulation (1 kHz 1–2 mA or 3 kHz 2 mA, 10 trials), initiated within 2 s of the onset of the sharp rise in bladder pressure signaling an imminent void, suppressed the void completely (**Figure 7B**). In the remaining rat, urine output was reduced by 50.8–59.0% (three trials) compared to the preceding spontaneous voids. Stimulating in between voids was tested in three rats and produced a very small transient increase in bladder pressure (0–4.2 mmHg, mean 1.6 mmHg), and an increase in tonic EMG activity of the EUS.

Gross examination of the abdominal cavity post mortem showed that the nerve cuff assembly had been encapsulated with connective tissue. There was no sign of pinching or twisting of the nerve. Additionally, the bladder catheter was still in place and no leakage was evident when the bladder was infused with saline containing Pontamine Sky Blue dye.

## Discussion

The aim of the present study was to assess in conscious rats the translational potential of the finding in anesthetized preparations that high frequency stimulation of the pelvic nerve could inhibit urinary voiding. In every animal tested, pelvic nerve stimulation inhibited imminent voids. The effect was rapid in onset, readily reversible and importantly, there was an absence of adverse side effects.

Laparotomy for chronic implantation of bladder cannula and electrodes on the pelvic nerve is by its nature invasive and involves a degree of surgical stress. However, our study showed that the procedure was well-tolerated by female Wistar rats. Within a few days post-operatively normal urine output had resumed and the rats displayed the characteristic diurnal fluctuation in voiding pattern with fewer, larger volume voids produced during the light period compared to the dark (Ranson et al., 2003; Herrera and Meredith, 2010; Negoro et al., 2013). Terminal experiments carried out several weeks post-operatively also indicated that the functionality of the electrode-nerve-bladder connection had not been compromised by long-term implantation of the electrode.

For convenience during urodynamic testing we chose to restrain our rats by confining them in a tube. Restraint is often used as a means to induce stress in rodents (Costa et al., 2005; Gameiro et al., 2006). However, rather than immobilizing them completely, the restrainer used in our study simply prevented the animals from turning. Moreover, they had been fully habituated to the environment and showed no signs of distress. Restraining rats in this way after prior habituation has been shown to result in marked lowering of circulating adrenocorticotrophic hormone levels compared to acutely restrained animals (Grissom et al., 2008). The incidence of movement-related artifacts on the cystometrogram is also greatly reduced in lightly restrained rats compared to freely moving animals (Morikawa et al., 1990). Importantly, in a comparative study no significant differences could be detected between urodynamic measures during cystometry carried out in freely moving or lightly restrained rats (Chen et al., 2016). Therefore we believe that mild restraint was not a confounding factor in our experiments.

Infusing saline into the bladder in conscious rats evoked repeated voids in line with previous reports (e.g., Schneider et al., 2015; Chen et al., 2016; Monjotin et al., 2017). For almost every void, the volume of fluid expelled exceeded the volume of saline infused into the bladder since the preceding void. This suggests that urine continued to drain into the bladder from the kidneys during cystometry, and that the bladder emptied fully during voids.

Stimulation of the pelvic nerve during the filling phase in between voids produced a transient increase in bladder pressure at stimulus onset. The time course of the rise in bladder pressure was much slower than the rise in pressure that occurred during spontaneous voids. In addition, a distinct contraction of the abdominal wall was clearly visible at the onset of stimulation. Therefore it seems likely that the nerve stimulation-evoked rise in bladder pressure was due to an increase in intra-abdominal pressure transmitted to the bladder rather than active contraction of the detrusor. In support of this idea, in terminal experiments in the same animals under anesthesia with the abdominal cavity open, high frequency stimulation of the pelvic nerve evoked only minimal changes in bladder pressure. However, further experiments in conscious rats with simultaneous recording of intra-abdominal and intra-vesicular pressure (Lee and Yoon, 2013) will be required to provide a definitive answer. Interestingly, despite the rise in bladder pressure, no urine escaped when stimulation was applied in between voids. In urethane-anesthetized rats undergoing continuous infusion cystometry we found that stimulating the pelvic nerve during the filling phase evoked a tonic contraction of the EUS (Crook and Lovick, 2017). It is likely that a similar response was evoked in conscious rats.

When the pelvic nerve stimulation was started immediately prior to an imminent void, it produced profound effects on voiding. In every rat we were able to either suppress voiding completely or to significantly reduce the volume of fluid voided. Effective stimulation frequencies and intensities were similar to those found to be optimal for suppressing micturition in anesthetized preparations (Crook and Lovick, 2017). As in anesthetized rats, there was a critical “window of opportunity” to begin stimulation following the onset of the sharp rise in bladder pressure, in order to inhibit voiding. Once past this “point of no return”, pelvic nerve stimulation appeared unable to modify the void.

Pelvic nerve stimulation was able to completely suppress, reduce or delay an imminent void in each rat we tested. Despite looking at a number of urodynamic and behavioral variables, we were unable to identify a factor that might determine the type of response that was evoked. However, one factor we did not take into account was the hormonal status of the rats. In female rats, estrous cycle has been shown to influence contractile responsiveness of the detrusor and to affect micturition pattern, at least in spontaneously hypertensive rats (Patra, 2013, wildtype animals not studied). Plasma vasopressin has also been shown to vary during the estrous cycle and will in turn, influence urine production and hence bladder filling (Forsling and Peysner, 1988). The estrous cycle stage may therefore have been influential in determining the outcome of pelvic nerve stimulation in different test sessions in the present study. Clearly, future studies in females should take the stage of the estrous cycle into account.

The mechanism by which high frequency stimulation of the pelvic nerve suppresses voiding is not yet understood. The pelvic nerve is a mixed nerve comprising small diameter myelinated and unmyelinated fibers (Hulsebosch and Coggeshall, 1982) around half of which are sensory afferents with conduction velocities in the Aδ and C fiber ranges (Shea et al., 2000; De Wachter, 2011). The remainder comprise largely parasympathetic preganglionic efferents and some postganglionic sympathetic fibers (Hulsebosch and Coggeshall, 1982). The effective stimulus frequencies (1–3 kHz) for inhibiting voiding, are below the frequencies that might be expected to induce nerve conduction block (>20 kHz; Zhao et al., 2014). However, it seems unlikely that the small nerve fibers would follow faithfully the 1–3 kHz stimulation we found to be effective in inhibiting voiding. Interestingly, the results of a recent modeling study suggest that stimulation in this frequency range would be more likely to activate the nerve in a manner that imposes a non-physiological pattern of firing (Pelot et al., 2017). The consequence of such afferent input may be a functional blockade of the micturition reflex circuitry at spinal or brainstem level so that the neural network is prevented from generating a co-ordinated void (Crook and Lovick, 2017). In an analogous manner, a non-physiological barrage of input to the pelvic ganglion via parasympathetic efferents in the nerve may produce a functional blockade of transmission to the postganglionic bladder efferent. In anesthetized rat preparations we found that whereas stimulation of the pelvic nerve at low frequencies <40 Hz evoked robust increases in bladder pressure, high frequency stimulation at the same intensity elicited only a small transient “on-response” consistent with failure of transmission through the ganglion (Crook and Lovick, 2017).

The effect of pelvic nerve stimulation on activity of the EUS is worthy of consideration. Whilst we did not measure activity in the EUS in conscious animals, we showed that in anesthetized rats undergoing continuous infusion cystometry high frequency pelvic nerve stimulation suppressed voiding accompanied by tonic contraction of the external sphincter. A similar effect in conscious rats, probably mediated by reflex activation of pudendal nerve efferents, would have helped to maintain continence during pelvic nerve stimulation.

The results highlight the powerful modulation of voiding that can be evoked by high frequency stimulation of the pelvic nerve in conscious rats. Urodynamic investigations in humans indicate that the appearance of the sensation of urge correlates with a sharp rise in bladder pressure (Oliver et al., 2003). In rats, initiating high frequency stimulation of the pelvic nerve at this time was able to suppress voiding. Importantly, there were no adverse behavioral effects and voiding resumed within minutes of stopping the stimulator. These findings raise the possibility of developing an alternative conditional neuromodulatory approach to UUI in humans in which high frequency stimulation of the pelvic nerve could be activated at the onset of urge sensation to suppress imminent voids. The facility to suppress or delay voiding on demand would give an individual with urge incontinence time to find a toilet, and so avoid the humiliating consequences of an incontinent episode. The present study was carried out in an animal model in which voiding is essentially normal, albeit more frequent than normal due to the high rate of bladder filling induced by continuous infusion cystometry. The next step toward translation would be to investigate effects of pelvic nerve stimulation in animal models of overactive bladder/urinary urge incontinence.

## Author Contributions

TL conceived the study and obtained funding with PI. CB carried out the bulk of the experimental work and analyzed the data, with input from TL and JC. TL prepared a draft of the manuscript with input from JC and CB. All authors approved the final version.

## Conflict of Interest Statement

The authors declare that the research was conducted in the absence of any commercial or financial relationships that could be construed as a potential conflict of interest.
